# Expanding the applicability of multiphoton fluorescence recovery after photobleaching by incorporating shear stress in laminar flow

**DOI:** 10.1117/1.JBO.28.7.076502

**Published:** 2023-07-21

**Authors:** Tresa M. Elias, Edward B. Brown, Edward B. Brown

**Affiliations:** a University of Rochester, Department of Biomedical Engineering, Rochester, New York, United States; b Manhattan College, Department of Physics, Riverdale, New York, United States

**Keywords:** diffusion, transport, flow, shear flow, fluorescence recovery after photobleaching

## Abstract

**Significance:**

Multi-photon fluorescence recovery after photobleaching (MPFRAP) is a nonlinear microscopy technique used to measure the diffusion coefficient of fluorescently tagged molecules in solution. Previous MPFRAP fitting models calculate the diffusion coefficient in systems with diffusion or diffusion in laminar flow.

**Aim:**

We propose an MPFRAP fitting model that accounts for shear stress in laminar flow, making it a more applicable technique for *in vitro* and *in vivo* studies involving diffusion.

**Approach:**

Fluorescence recovery curves are generated using high-throughput molecular dynamics simulations and then fit to all three models (diffusion, diffusion and flow, and diffusion and shear flow) to define the limits within which accurate diffusion coefficients are produced. Diffusion is simulated as a random walk with a variable horizontal bias to account for shear flow.

**Results:**

Contour maps of the accuracy of the fitted diffusion coefficient as a function of scaled velocity and scaled shear rate show the parameter space within which each model produces accurate diffusion coefficients; the shear-flow model covers a larger area than the previous models.

**Conclusion:**

The shear-flow model allows MPFRAP to be a viable optical tool for studying more biophysical systems than previous models.

## 1 Introduction

Multiphoton fluorescence recovery after photobleaching (MPFRAP) is a microscopy technique used to probe the local mobility and/or measure the diffusion coefficient of fluorescently tagged macromolecules in living tissues.^1^^–^^6^ In the original “point” MPFRAP method, a stationary, high-intensity, mode-locked laser is briefly flashed within a region of interest to generate photobleaching of fluorescent molecules. The laser is then attenuated to a lower intensity to monitor still-fluorescent molecules diffusing into the region. This results in a fluorescence recovery as a function of time curve, which can be fitted to an analytical equation from which a diffusion coefficient can be extracted. Utilizing multiphoton excitation allows for the assessment of diffusion coefficients in a three-dimensionally resolved volume. Useful variations of this original “point” MPFRAP exist whereby lines or other bleach patterns are generated, and diffusion coefficients or simple recovery times are calculated.^7^^–^^9^ However, “point” MPFRAP is the technique we are referring to as “MPFRAP” in this work.

The original MPFRAP model calculates diffusion coefficients in systems only governed by diffusion, i.e., fluorescent molecules freely diffusing in solution,^1^ termed the diffusion-only model. To make this technique more applicable to *in vivo* systems, the original MPFRAP model was adapted to extract diffusion coefficients in systems with flow, termed the diffusion-convection model.^10^ In the study described here, we further expand the applicability of the current MPFRAP models by incorporating shear stress in the presence of laminar flow, i.e., the flow speed in the x direction is a linear function of position along the z, or optical, axis, termed the shear flow model. This model accounts for the shear forces that play a critical role in modeling capillary flow, drug delivery in complex microenvironments, microfluidics, and other biological and physical systems.^11^^,^^12^ Studies have shown that shear forces induce signaling in many physiological processes, such as inflammation, arterial thrombus formation, acute stroke, and atherogenesis.^13^^–^^16^ The model in this study accounts for shear flow when it determines diffusion coefficients, thus improving the efficacy and applicability of MPFRAP in testing biomaterials and drug delivery, investigating microfluidics, and *in vivo* applications. Note that this study specifically focuses on measuring diffusion coefficients in the presence of shear flow, not on measuring parameters of the flow field itself: there are ways of measuring flow speed and shear rate other than via MPFRAP (for example via line scans of fluorescent beads on a multiphoton-laser scanning microscope) that are often better than MPFRAP because they offer directional information.

To model fluorescence recovery in the presence of various combinations of flow and shear stress, we performed molecular dynamics simulations in a high-throughput manner by utilizing parallel computing in MATLAB (The MathWorks, Natick, Massachusetts, United States). This produced a simulated concentration-versus-time distribution from which we calculated a fluorescence-versus-time distribution, which was then fit to various MPFRAP models. We then determined at what combinations of flow and shear stress our previous models failed to compute the correct diffusion coefficient, and then evaluated the success of our new model at these combinations. We then evaluated the fitting models using *in vitro* MPFRAP data collected in microfluidic channels that induce various combinations of velocity and shear stress.

## 2 Materials and Methods

### 2.1 Theoretical Derivation of the Shear Flow MPFRAP Model

The initial concentration distribution of unbleached fluorophores immediately following a photobleaching pulse is given by Brown et al.^1^

$$c(x,y,z;t=0)=c_{o}\;\exp[-(1/b)q_{b}\delta_{b}\langle I_{bl}^{b}(x,y,z)\rangle \Delta t],$$ (1)

where $c_{o}$ is the initial equilibrium concentration of the fluorophore; b is the number of photons absorbed per photobleaching event; $q_{b}$ is the quantum efficiency for b-photon photobleaching; $\delta_{b}$ is the multiphoton fluorescence action cross-section of the fluorophore for the order of excitation required for photobleaching; $\left\langle I_{bl}^{b}(x,y,z)\right\rangle$ is the time average of the bleach intensity to the b’th power; and Δt is the bleaching pulse duration. This distribution assumes that Δt is significantly faster than any transport time in the system. In this work that means that Δt is significantly faster than the recovery times due to diffusion, due to average flow speed, and due to shear rate.

The bleach intensity can be represented as a 3D Gaussian that is a function of the time average of the intensity at the two-photon focal volume center raised to the b’th power, and the $1/e^{2}$ radial and axial dimensions of the two-photon focal volume, $\omega_{r}$ and $\omega_{z}$, respectively:^1^

$$\langle I_{bl}^{b}(x,y,z)\rangle =\langle I_{bl}^{b}(0,0,0)\rangle \exp[-\frac{2b(x^{2}+y^{2})}{\omega_{r}^{2}}-\frac{2bz^{2}}{\omega_{z}^{2}}].$$ (2)

Assuming a properly overfilled back aperture, the radial and axial dimensions of a two-photon focal volume are defined as $\omega_{r}\equiv 2.6\lambda /(2\pi \mathrm{NA})$ and $\omega_{z}\equiv 8.8n\lambda /[2\pi {\left(\mathrm{NA}\right)}^{2}]$, respectively, where λ is the wavelength of the excitation laser, n is the index of refraction of the immersion media, and NA is the numerical aperture of the lens.^17^

The fluorescence intensity at time t generated by a stationary weak monitoring beam that produces fluorescence through an m-photon process is given as^1^

$$F(t)=\frac{\delta_{m}E}{m}\int \langle I_{mo}^{m}(x,y,z)\rangle c(x,y,z;t)dx\;dy\;dz,$$ (3)

where $\delta_{m}$ is the multiphoton fluorescence action cross-section of the fluorophore for the order of excitation required to produce fluorescence, E is the collection efficiency of the detection system, and m is the number of photons absorbed per excitation event. $I_{mo}^{m}$ is a Gaussian intensity distribution as in Eq. (2). Therefore, to derive F(t) for any mode of fluorescence recovery (i.e., diffusion only, diffusion, flow, etc.) one must determine c(x,y,z,t), then input that into Eq. (3) to find F(t).

The original MPFRAP model assumes that fluorescence recovery is due to diffusion only. The concentration profile, c(x,y,z,t), is determined using the diffusion equation and the resultant expression for fluorescence recovery is then

$$F(t)=F_{o}\overset{\infty }{\underset{n=0}{\sum }}\frac{{\left(-\beta \right)}^{n}}{n!}\frac{1}{\left(1+n+\frac{2nt}{\tau_{D}}\right)}\frac{1}{{\left(1+n+\frac{2nt}{R\tau_{D}}\right)}^{1/2}},$$ (4)

where $\tau_{D}$ is the characteristic recovery time due to diffusion and R is the square of the ratio of axial to radial $1/e^{2}$ dimensions of the focal volume. In this original model, the two fitting parameters are β and $\tau_{D}$. The bleach depth parameter β is defined as $\beta \equiv (1/b)q_{b}\delta_{b}\langle I_{bl}^{b}(0,0,0)\Delta t\rangle$. The diffusion coefficient can be calculated from the characteristic recovery time with the relationship $D=\omega_{r}^{2}/8\tau_{D}$. To expand the range of systems where one can measure accurate diffusion coefficients, Sullivan et al. incorporated convective flow with diffusion into a fluorescence recovery model.^10^ In the case of one-dimensional flow parallel to the imaging plane, the fluorescence recovery can be modeled using the following equation:

$$F(t)=F_{o}\overset{\infty }{\underset{n=0}{\sum }}\frac{{\left(-\beta \right)}^{n}}{n!}\frac{\exp[-\frac{4n{\left(\frac{t}{\tau_{v}}\right)}^{2}}{1+n+\frac{2nt}{\tau_{D}}}]}{(1+n+\frac{2nt}{\tau_{D}}){\left(1+n+\frac{2nt}{R\tau_{D}}\right)}^{1/2}}.$$ (5)

This model introduces a third fitting parameter, which describes the characteristic recovery time due to flow, $\tau_{v}$. The flow speed in this model can be calculated by the relationship $v=\omega_{r}/\tau_{v}$.

The previous model assumes that flow is uniform, but many biological and experimental systems are dominated by shear flow whereby the flow speed in one direction (i.e., the x direction) varies with position in another direction (i.e., the z direction). To make MPFRAP applicable to more *in vivo* systems, we will therefore incorporate shear flow in the convection element by adding a time-and position-dependent coordinate shift to model shear along the axis perpendicular to flow, before the mathematical convolution of the concentration profile with the excitation laser profile.

To begin the derivation, consider the time-dependent concentration profile of unbleached fluorophores evolving only due to diffusion, as derived in Ref. 1 but expressed here in Cartesian coordinates

$$c(x,y,z;t)=\overset{\infty }{\underset{n=0}{\sum }}A_{n}(t)e^{-\mu_{n}(t)x^{2}}e^{-\mu_{n}(t)y^{2}}e^{-\upsilon_{n}(t)z^{2}},$$ (6)

where

$$A_{n}(t)=c_{o}\frac{{\left(-\beta \right)}^{n}}{n!}\frac{1}{(1+\frac{8bnDt}{\omega_{r}^{2}}){\left(1+\frac{8bnDt}{\omega_{z}^{2}}\right)}^{1/2}},$$ (7)

$$\mu_{n}(t)=\frac{2bn}{\omega_{r}^{2}}\frac{1}{\left(1+\frac{8bnDt}{\omega_{r}^{2}}\right)},$$ (8)

$$\upsilon_{n}(t)=\frac{2bn}{\omega_{z}^{2}}\frac{1}{\left(1+\frac{8bnDt}{\omega_{z}^{2}}\right)}.$$ (9)

To incorporate flow in the presence of shear stress, we apply a time-dependent coordinate shift to this distribution representing flow along the x-axis which varies with z. In the frame of reference of the observer, we define $x^{\prime }=x+v_{x}t$, $y^{\prime }=y$, and $z^{\prime }=z$. Here $v_{x}=v_{o}+\gamma z^{\prime }$ where γ is the shear rate and $v_{o}$ is the flow speed in the x-direction at the center of the focal volume. The time-dependent fluorophore concentration now becomes

$$c(x^{\prime },y^{\prime },z^{\prime };t)=\overset{\infty }{\underset{n=0}{\sum }}A_{n}(t)e^{-\mu_{n}(t){\left(x^{\prime }-v_{x}t\right)}^{2}}e^{-\mu_{n}(t)y^{\prime 2}}e^{-\upsilon_{n}(t)z^{\prime 2}}.$$ (10)

By substituting Eq. (10) into Eq. (3), we now have

$$F(t)=\frac{\delta_{m}E}{m}\langle I_{o}\rangle \overset{\infty }{\underset{n=0}{\sum }}A_{n}(t)\int_{-\infty }^{+\infty }e^{-\mu_{n}(t){\left(x^{\prime }-v_{x}t\right)}^{2}-(\frac{2m}{\omega_{r}^{2}})x^{\prime 2}}dx^{\prime }\;\times \int_{-\infty }^{+\infty }e^{-(\mu_{n}(t)+\frac{2m}{\omega_{r}^{2}})y^{\prime 2}}dy^{\prime }\int_{-\infty }^{+\infty }e^{-(\upsilon_{n}(t)+\frac{2m}{\omega_{z}^{2}})z^{\prime 2}}dz^{\prime }.$$ (11)

Since $v_{x}$ is a function of $z^{\prime }$, we must separate and rearrange the terms in the $x^{\prime }$ and $z^{\prime }$ integrals. We will define J(t) as the product of the three integrals in Eq. (11), and we begin by solving the $y^{\prime }$ integral using the integral identity $\int_{-\infty }^{+\infty }e^{-\rho^{2}x^{2}}dx=\sqrt{\pi }/\rho (\rho >0)$. Now, J(t) becomes

$$J(t)={\left[\frac{\pi }{\mu_{n}(t)+2m/\omega_{r}^{2}}\right]}^{\frac{1}{2}}\int_{-\infty }^{+\infty }e^{-\mu_{n}(t){\left(x^{\prime }-v_{x}t\right)}^{2}-(\frac{2m}{\omega_{r}^{2}})x^{\prime 2}}dx^{\prime }\int_{-\infty }^{+\infty }e^{-(\upsilon_{n}(t)+\frac{2m}{\omega_{z}^{2}})z^{\prime 2}}dz^{\prime }.$$ (12)

To begin integrating the $x^{\prime }$ integral we first define $v(z^{\prime })=v_{0}+\gamma z^{\prime }$, then define $u^{\prime }(x^{\prime }z^{\prime })$ with Eq. (13) and observe that $du^{\prime }/dx^{\prime }=1$; this will allow us to make a variable substitution to convert $dx^{\prime }$ to $du^{\prime }$ as shown in Eq. (14)

$$u^{\prime }(x^{\prime }z^{\prime })=x^{\prime }-\frac{\mu_{n}(t)v(z^{\prime })t}{\mu_{n}(t)+2m/\omega_{r}^{2}}.$$ (13)

Plugging in the $u^{\prime }(x^{\prime }z^{\prime })$ and rearranging the terms such that all solely $z^{\prime }$-dependent terms are in the $z^{\prime }$ integral, J(t) now becomes

$$J(t)={\left[\frac{\pi }{\mu_{n}(t)+2m/\omega_{r}^{2}}\right]}^{\frac{1}{2}}\int_{-\infty }^{+\infty }e^{-(\mu_{n}(t)+\frac{2m}{\omega_{r}^{2}})u^{\prime }(x^{\prime }z^{\prime }{)}^{2}}du^{\prime }\;\times \int_{-\infty }^{+\infty }e^{-[\frac{2m\mu_{n}(t)v(z^{\prime }{)}^{2}t^{2}}{\mu_{n}(t)\omega_{r}^{2}+2m}+(\upsilon_{n}(t)+\frac{2m}{\omega_{z}^{2}})z^{\prime 2}]}dz^{\prime }.$$ (14)

Now the $u^{\prime }$ integral is in the same form as the previous $y^{\prime }$ integral, so we can apply the same integral identity to now obtain a simplified version of J(t) with only the $z^{\prime }$ integral remaining

$$J(t)=\frac{\pi }{\mu_{n}(t)+2m/\omega_{r}^{2}}\int_{-\infty }^{+\infty }e^{-[\frac{2m\mu_{n}(t)(v_{o}+\gamma z^{\prime }{)}^{2}t^{2}}{\mu_{n}(t)\omega_{r}^{2}+2m}+(\upsilon_{n}(t)+\frac{2m}{\omega_{z}^{2}})z^{\prime 2}]}dz^{\prime }.$$ (15)

Through rearranging and collecting terms and performing integration with the integral identity $\int_{-\infty }^{+\infty }e^{-(ax^{2}+bx+c)}dx=\sqrt{\frac{\pi }{a}}e^{(b^{2}-4ac)4a}$, we obtain a final form of J(t),

$$J(t)=[\frac{\pi }{\mu_{n}(t)+\frac{2m}{\omega_{r}^{2}}}]{\left[\frac{\pi }{\upsilon_{n}(t)+\frac{2m}{\omega_{z}^{2}}+f_{o}\mu_{n}(t)\gamma^{2}t^{2}}\right]}^{\frac{1}{2}}\;\times \exp[-f_{o}\mu_{n}(t)v_{o}^{2}t^{2}[1-\frac{f_{o}\gamma^{2}\mu_{n}(t)t^{2}}{\upsilon_{n}(t)+\frac{2m}{\omega_{z}^{2}}+f_{o}\mu_{n}(t)\gamma^{2}t^{2}}]],$$ (16)

where

$$f_{o}=\frac{2m/\omega_{r}^{2}}{\mu_{n}(t)+2m/\omega_{r}^{2}}.$$ (17)

We can plug this expression for J(t) back into Eq. (11) for the three integrals, and then define $F_{o}$, the prebleach fluorescence, using the following equation:

$$F_{o}=c_{o}\frac{\delta_{m}E}{m}\langle I_{o}\rangle \frac{\pi }{2m/\omega_{r}^{2}}{\left[\frac{\pi }{2m/\omega_{z}^{2}}\right]}^{\frac{1}{2}}.$$ (18)

We now introduce our recovery time variables using the definitions: $\tau_{D}=\omega_{r}^{2}/8D$, $\tau_{v}=\omega_{r}/v_{o}$, and $\tau_{\gamma }=1/\gamma$. Recalling the fact that MPFRAP is usually performed with two-photon processes, we plug in m=b=2 to further simplify, and we also recall that $R=\omega_{z}^{2}/\omega_{r}^{2}$. We now have the final fluorescence recovery equation that accounts for shear stress in the presence of laminar flow

$$F(t)=F_{o}\sum_{n=0}^{\infty }A_{n}(t)B_{n}(t)S_{n}(t),$$

where

$$A_{n}(t)=\frac{(-\beta {)}^{n}}{n!}\frac{1}{\left(1+\frac{2nt}{\tau_{D}}\right)}\frac{1}{(1+\frac{2nt}{R\tau_{D}}{)}^{1/2\;}}\;B_{n}(t)=\frac{1}{\frac{n}{1+2nt/\tau_{D}}+1}\;\frac{1}{{\left[1+\frac{n}{1+\frac{2nt}{R\tau_{D}}}+\frac{nR{\left(\frac{t}{\tau_{\gamma }}\right)}^{2}}{1+n+\frac{2nt}{\tau_{D}}}\right]}^{1/2\;}}\;\;S_{n}(t)=\exp[\frac{-4n{\left(\frac{t}{\tau_{v}}\right)}^{2}}{1+n+\frac{2nt}{\tau_{D}}}(1-\frac{\frac{nR{\left(\frac{t}{\tau_{\gamma }}\right)}^{2}}{1+n+2nt/\tau_{D}}}{1+\frac{n}{1+2nt/R\tau_{D}}+\frac{nR{\left(\frac{t}{\tau_{\gamma }}\right)}^{2}}{1+n+2nt/\tau_{D}}})]$$ (19)

For simplicity, the exponential term in J(t) from Eq. (16) was named $S_{n}(t)$. Note that in the case where there is no shear stress ($\tau_{\gamma }\to \infty$) this model reduces to the diffusion-convection model given by Eq. (5). In the case where there is no flow $\left(\tau_{v}\to \infty \right)$ and no shear stress ($\tau_{\gamma }\to \infty$), this model reduces to the diffusion only model, given by Eq. (4).

**Fig. 1:**
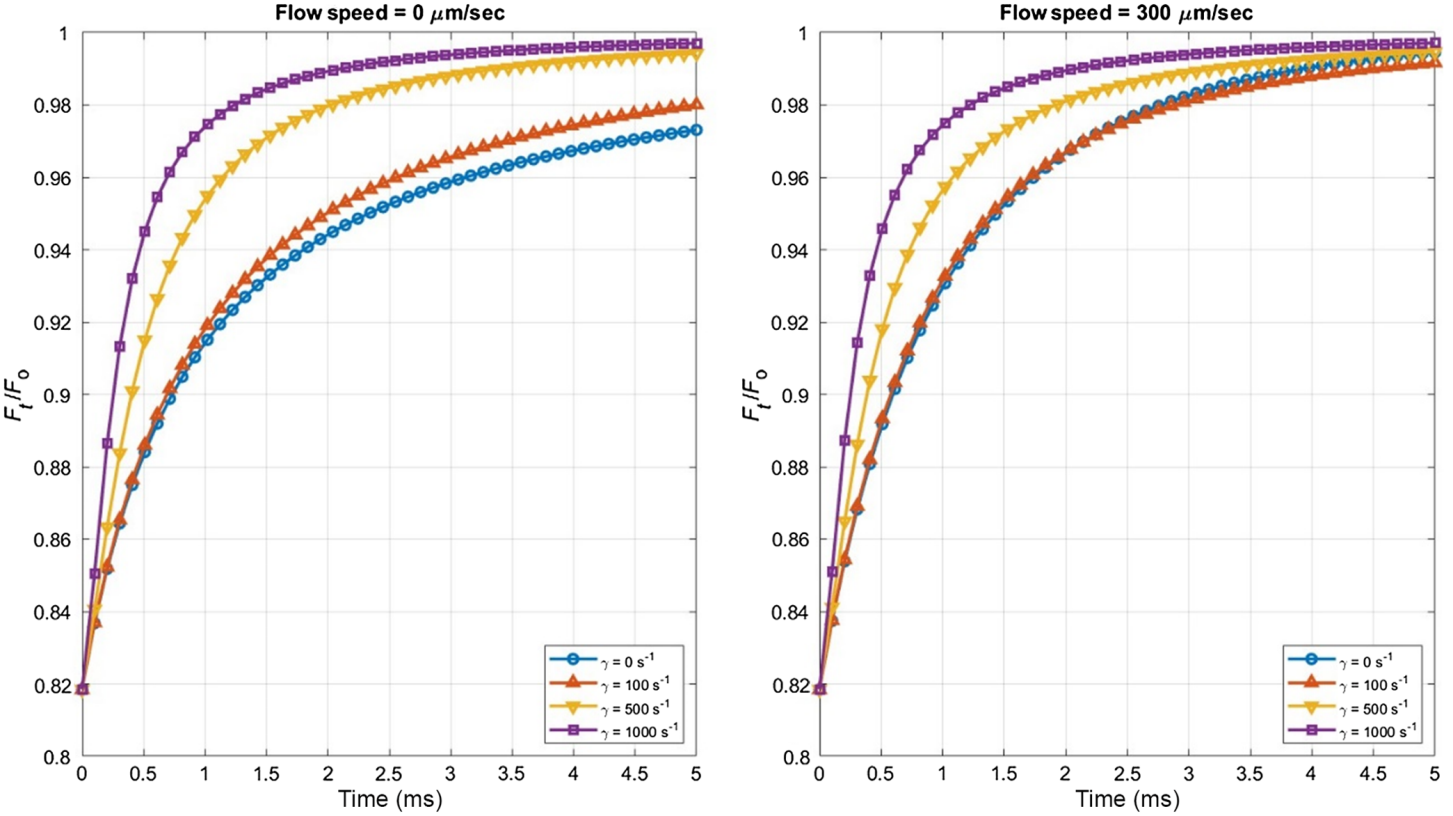
Mathematical modeling of MPFRAP with shear flow. Theoretical MPFRAP curves calculated at various stress rates using the shear flow model Eq. (21) where $D=60\;\mu m^{2}/s$, and $v_{o}=0$ (a) and $v_{o}=300\;\mu m/s$ (b).

Figure 1 shows the predicted fluorescence recovery curves using the new shear flow model derived above, with typical values of β=0.6 and $D=60\;\mu m^{2}/s$. In Fig. 1(a), we observe fluorescence recovery curves in the absence of flow at the center of the focal volume ($v_{o}=0$) (reminiscent of a “whirlpool” exactly centered on the focal volume), at four different shear rates. Figure 1(b) shows a more realistic scenario with non-zero flow speed at the center of the focal volume. As expected, the presence of shear tends to accelerate recovery of fluorescence.

### 2.2 Monte Carlo Simulation

To generate artificial data upon which to test our new model, we simulated the motion of bleached molecules under different diffusion/flow/shear conditions. As in previous MPFRAP simulations,^10^^,^^17^ space was discretized into a regular lattice structure with spacing defined by the expected diffusive properties and diffusion was modeled as a random walk on this 3D lattice.^18^^,^^19^ The lattice spacing was determined by the 3D diffusion equation $\langle r^{2}\rangle =6Dt$, where the diffusion coefficient, D, was chosen *a priori* and t represents the time step. For this study, D was chosen to be $60\;\mu m^{2}/s$, which is a typical diffusion coefficient for fluorescein isothiocyanate (FITC)-bovine serum albumin (BSA) at room temperature. The bleach depth parameter was set to 0.6.

Previously,^10^^,^^17^ the time step was set equal to 1/1000 of the typical diffusive recovery time for a system with a diffusion coefficient D and with radial and axial focal volume dimensions $\omega_{r}$ and $\omega_{z}$, thus $t_{\text{step}}=\tau_{D}/1000$.^10^^,^^17^ However, for certain combinations of high flow and large shear rate, the half recovery time of the curve may be smaller than $\tau_{D}$, meaning that the time step would be too large to accurately simulate a fluorescence recovery curve. Instead, we account for all possible recovery mechanisms by calculating the time step as 1/1000 of the expected half recovery time of the curve, calculated using Eq. (20) below. Defining the time step as 1/1000 of the half recovery time due to all possible recovery mechanisms instead of 1/1000 of the half recovery time due to just diffusion^10^^,^^17^ will ensure a small enough time step to accurately simulate a fluorescence recovery curve at all combinations of flow and shear rate

$$\frac{1}{\tau_{\frac{1}{2}}}=\frac{1}{\tau_{D}}+\frac{1}{\tau_{v}}+\frac{1}{\tau_{y}}.$$ (20)

To start the simulation, this lattice was populated with one candidate molecule at every lattice point, creating a uniform distribution of bleached fluorophores, spanning from $-2\omega_{r}$ to $2\omega_{r}$ in the x- and y-directions, and $-2\omega_{z}$ to $2\omega_{z}$ in the z-direction. In our simulations, the NA was 0.8 and hence $\omega_{r}=0.404\;\mu m$ and $\omega_{z}=2.27\;\mu m$. Next, a “probability threshold” for each candidate molecule was calculated using Eq. (21) below, which is obtained by substituting Eq. (2) into Eq. (1), setting b=2 for a two-photon bleaching process, and $c_{o}=1$, and represents a distribution of the appropriate shape for a given bleach depth parameter β. Each candidate molecule was assigned a random number between zero and one, using a uniformly distributed random number generator. The candidate was eliminated if the random number was above the threshold. This process was repeated until there were 20,000 bleached molecules on the lattice. There were no constraints on how many molecules could be at a single node.

$$p_{bl}(x,y,z;t=0)=1-\exp\{-\beta \;\exp[-\frac{4(x^{2}+y^{2})}{\omega_{r}^{2}}-\frac{4z^{2}}{\omega_{z}^{2}}]\}.$$ (21)

Once the initial distribution was created, all molecules were allowed to take one step in a random direction. A single molecule can move in one of six directions; either the positive or negative x-direction, positive or negative y-direction, or positive or negative z-direction. Thus, each molecule was assigned a random integer from 1 to 6, using a uniformly distributed random number generator, where each integer is assigned to one of the six possible directions. The length of each of these steps is defined as $L=\sqrt{6Dt}$. To simulate shear flow, each molecule experienced a horizontal displacement in the x-direction after each step, defined as $x_{\text{bias}}=(v_{x}+\gamma z)t$, where $v_{x}$ is the flow speed at z=0; γ is the shear rate; z is the z-coordinate of the particle after its random step of length L; and t is the time between each step. Once all molecules have taken their random step and have been horizontally displaced, the fluorescence signal from the distribution of molecules at that time step is calculated.

In a physical MPFRAP experiment, the fluorescence of unbleached molecules is monitored; however, simulating an infinitely large volume of molecules is less feasible and more computationally expensive in simulation. For this reason, we are simulating a finite number of bleached fluorophores. Since we are monitoring the bleached fluorophores, we need to calculate the “missing fluorescence” that these fluorophores would have produced had they not been bleached. We can then use the missing fluorescence to determine the fluorescence of the remaining unbleached molecules, which will be used to generate the MPFRAP curve.

Calculating the missing fluorescence of the unbleached molecules can be done by re-expressing the integral in Eq. (3) as a sum of monitor intensity, given by Eq. (2) with b→m, over all bleached fluorophore locations $\left(x_{i},y_{i},z_{i}\right)$, yielding

$$F_{bl}(t)=\frac{\delta_{m}E}{m}\underset{i}{\sum }\;\exp[-\frac{2b(x_{i}^{2}+y_{i}^{2})}{\omega_{r}^{2}}-\frac{2bz_{i}^{2}}{\omega_{z}^{2}}].$$ (22)

Now that we have the missing fluorescence of the bleached fluorophores, we obtain the fluorescence of the unbleached fluorophores, F(t), by recognizing that at any given time after the bleaching event, the fluorescence of the unbleached molecules will be equal to the difference between the prebleach fluorescence ($F_{o}$) and the missing fluorescence of the bleached molecules, thus $F(t)=F_{o}-F_{bl}(t)$. Therefore, to produce F(t) from $F_{bl}(t)$, we first need to deduce $F_{o}$ from only the information carried in the $F_{bl}(t)$ curve. We can deduce $F_{o}$ specifically from $F_{bl}(0)$ by first taking the t=0 limit of Eq. (19),

$$F(0)=F_{0}\overset{\infty }{\underset{n=0}{\sum }}\frac{(-\beta {)}^{n}}{n!}\frac{1}{{\left(1+n\right)}^{3/2}}.$$ (23)

And substituting this into the t=0 expression $F(0)=F_{o}-F_{bl}(0)$, then solving for $F_{o}$:

$$F_{o}=\frac{F_{bl}(0)}{1-\sum_{n=0}^{\infty }\frac{{\left(-\beta \right)}^{n}}{n!}\frac{1}{{\left(1+n\right)}^{3/2}}}.$$ (24)

**Fig. 2:**
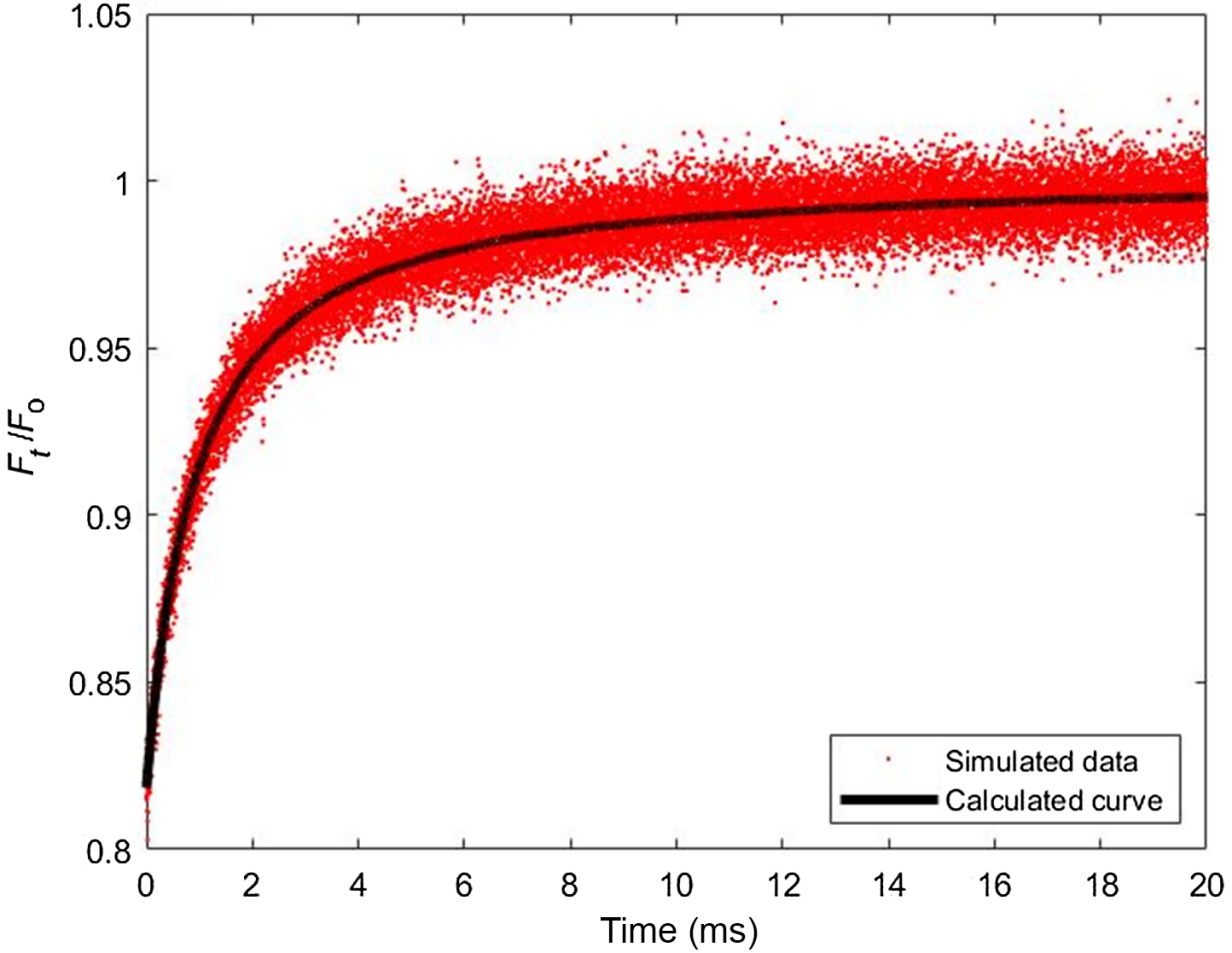
Example Monte Carlo simulated recovery curve. Fluorescence recovery curve generated with MPFRAP molecular dynamics algorithm in the presence of shear flow. Data are shown with the addition of 3% relative noise, and overlayed is the calculated curve from the mathematical model in Eq. (19).

Once the fluorescence recovery curve F(t) is simulated, additional noise is introduced to the curve to represent experimental *in vitro* and *in vivo* MPFRAP curves more closely.^1^^,^^10^^,^^17^ Specifically, the poissrnd() function in MATLAB was used to generate a Poisson distributed random number with mean of one, and a distribution width of ∼0.03. This random number was multiplied by each F(t) data point, producing final simulated FRAP curves with a relative noise fraction of 3%, which closely mimics the relative noise found in *in vitro* experiments (see Fig. 2).

The simulated fluorescence recovery curves are then fit to the three fitting models (diffusion-only, diffusion-convection, and shear flow) to extract the diffusion coefficient. A typical simulated fluorescence recovery curve is shown in Fig. 2, with the addition of 3% noise and the corresponding theoretical curve using the shear flow model.

### 2.3 Fitting Algorithm and Data Analysis

Simulated data were fit to the three fitting models using the Levenberg-Marquardt least squares fitting method implemented in MATLAB (The MathWorks, Natick, Massachusetts, United States) using the lsqcurvefit function. This function requires an initial guess for each fitting parameter as an input. These seed values were calculated as follows.

First, a seed value for the bleach depth parameter β was determined by evaluating the shear-flow MPFRAP equation [Eq. (19)] at t=0 and comparing it with the first data point of the fluorescence recovery curve after photobleaching, F(0). The seed value of β is the value of β that satisfies Eq. (25) for a given recovery curve, where N=10 for computational efficiency.

$$F(0)=F_{0}\overset{N}{\underset{n=0}{\sum }}\frac{{\left(-\beta_{\text{seed}}\right)}^{n}}{(n+1)!\sqrt{n+1}}.$$ (25)

Next, we generate seed values for $\tau_{D}$, $\tau_{v}$, and $\tau_{\gamma }$. For each term we do this by assuming that the recovery is dominated entirely by that process. Starting with the seed value for $\tau_{D}$, we assume all recovery is due to diffusion. In an automated fashion, we obtain the data point that is closest to or at the half-recovery fluorescence value and define that corresponding time as $\tau_{H}$. The seed value for $\tau_{D}$ is defined as the half-recovery time of the MP-FRAP curve, $\tau_{H}$.

The seed value for $\tau_{v}$ is generated by assuming that recovery is only due to flow. In this case, we take the limits $\tau_{D}\to \infty$ and $\tau_{\gamma }\to \infty$ in Eq. (19), which leaves us with an expression for the flow-dominated fluorescence recovery:

$$\frac{F(t)}{F_{o}}=\overset{\infty }{\underset{n=0}{\sum }}\frac{{\left(-\beta \right)}^{n}}{n!}\frac{\exp[\frac{-4n{\left(\frac{t}{\tau_{v}}\right)}^{2}}{1+n}]}{{\left(1+n\right)}^{3/2}}.$$ (26)

We then make the substitution $x^{\prime }=(t/\tau_{v}{)}^{2}$ and plot $F(x^{\prime })/F_{o}$ as a function of $x^{\prime }$. The seed value for $\tau_{v}$ is then obtained by finding the half-recovery time of this curve, which is denoted $x_{1/2}^{\prime }$. This value was determined to be $x_{1/2}^{\prime }=0.3625$. Plugging back in for $x^{\prime }$, we can solve for an expression for the seed value of $\tau_{v}$ as follows:

$$\tau_{v_{\mathrm{seed}}}=\frac{\tau_{H}}{\sqrt{0.3625}}.$$ (27)

Last, we determine a seed value for $\tau_{\gamma }$ by taking the limits $\tau_{D}\to \infty$ and $\tau_{v}\to \infty$ in Eq. (19), which leaves us with an expression for shear flow dominated fluorescence recovery:

$$\frac{F(t)}{F_{o}}=\overset{\infty }{\underset{n=0}{\sum }}\frac{{\left(-\beta \right)}^{n}}{n!}\frac{1}{(1+n)\sqrt{1+n+\frac{nR{\left(\frac{t}{\tau_{\gamma }}\right)}^{2}}{1+n}}}.\;$$ (28)

Making a similar substitution and using the same mathematical steps we used to calculate $\tau_{v_{\text{seed}}}$, we see that the shear flow dominated $x_{1/2}^{\prime }=0.145$. Plugging back in for $x^{\prime }$, we can solve for an expression for the seed value for $\tau_{\gamma }$ as follows:

$$\tau_{\gamma_{\text{seed}}}=\frac{\tau_{H}}{\sqrt{0.145}}.$$ (29)

These seed values are used as the initial guesses that are a required input of the lsqcurvefit function in MATLAB. An optional input of the lsqcurvefit function is a lower and upper bound for the fitting parameters which imposes bounds that these parameters cannot exceed. While one can imagine many experimental situations where biological or physical insight can provide useful bounds on these parameters, to remain unbiased and test the authenticity of our fitting models in a “worst case scenario,” initially we did not impose any bounds on the fitting parameters.

Simulation and fitting algorithms were run on a BlueHive supercomputer equipped with multiple CPU cores and nodes (Center for Integrated Research Computing, University of Rochester, Rochester, New York, United States). Utilizing high-throughput parallel computing allowed us to generate artificial MPFRAP data and fit to each of the three models for many different combinations of input diffusion coefficients, velocities, and shear rates. Simulations were run at 27 different flow speeds and 64 different shear rates, with 20 repetitions at each point, resulting in a total of 34,560 simulations. All software for simulating MPFRAP data and fitting that data are publicly available on Code Ocean: https://doi.org/10.24433/CO.4821156.v1.

### 2.4 *In vitro* MPFRAP with Shear Flow

**Fig. 3:**
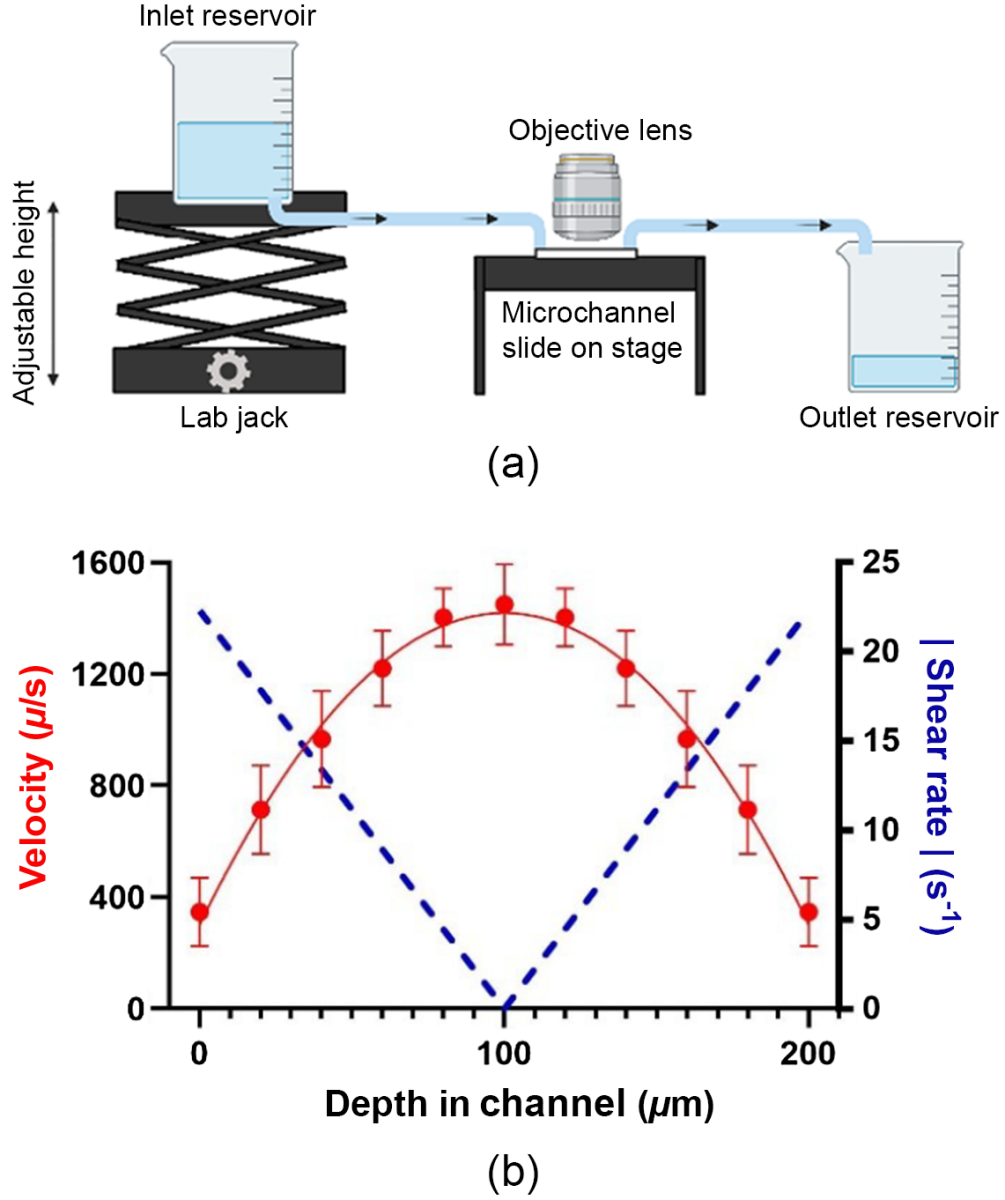
Experimental set-up for MPFRAP with shear flow and velocity and shear profile within a 200  μm microchannel. (a) Schematic diagram of experimental set-up for MPFRAP with shear flow. (b) Velocity measurements were computed from line scan images of fluorescent beads. Data are shown as mean ± standard deviation (n≥15). Velocity data were fit to a second-order polynomial and shear rate data were obtained by taking the absolute value of the derivative of the velocity profile.

We also evaluated the three fitting models in an *in vitro* system with various combinations of velocity and shear rate. This system used a microfluidic channel with a height of 200  μm, length of 50 mm, and width of 5 mm (μ-slide I Luer, Ibidi). To initiate gravity-driven flow through the channel, a large inlet reservoir was placed on an adjustable lab jack to control the height of the reservoir relative to the channel. The outlet reservoir was kept at a constant height. The inlet reservoir was filled with 300 mL of a 1  mg/mL solution of FITC conjugated to 2000 kDa dextran (Sigma). To this solution we added 1  μL/mL red fluorescent microspheres (FluoSpheres, Molecular Probes/Invitrogen). The velocity of the bead/dye solution through the channel was manipulated by adjusting the height of the inlet reservoir and quantified by taking line scan images of the fluorescent beads, which were then analyzed in ImageJ. Three line scan images were taken prior to 3 MPFRAP measurements at a specific location within the channel, yielding at least 15 lines at each location. The measured velocities were plotted as a function of height within the channel and fit to a second-order polynomial, as seen in Fig. 3. The shear rate as a function of height within the channel was then calculated as the derivative of the flow profile. This produced a velocity and shear-rate at each location where a MPFRAP measurement was made, which was then converted to a scaled velocity ($v_{s}$) and scaled shear rate ($\gamma_{s}$) using the focal volume dimensions of the lens.

A mode-locked Ti-Sapphire laser (Mai Tai; Spectra Physics, Mountain View, California, United States) delivering laser light with 80-fs pulses at a repetition rate of 100 MHz. Rapid modulation between bleach and monitor laser intensities was achieved with a KDP* Pockels Cell (model No. 350-80; Conoptics, Danbury, Connecticut, United States). Timing of the bleach and monitor pulses was delivered by a pulse generator (model No. DG535, Stanford Research Systems, Sunnyvale, California, United States). Simultaneously, the voltage output to the Pockels Cell was set and switched by a custom control box. After the Pockels Cell, the laser was directed through an Olympus Fluoview 300 laser-scanning microscope to the back aperture of a 0.8 NA, 40× water immersion objective lens (Olympus, Center Valley, Pennsylvania, United States). The back aperture of the objective was overfilled, yielding a focal volume with $1/e^{2}$ radius of 0.403  μm in the radial direction and 2.27  μm in the axial direction.^10^ The objective lens focused the focal volume at various positions within the microfluidic channel. The fluorescence emission was separated from the excitation light by a short-pass dichroic mirror (Chroma Technologies, Brattleboro, Vermont, United States). When imaging fluorescent beads to determine flow properties within the microfluidic channel, the emission signal was filtered by a 605/35 emission filter (Chroma Technologies, Brattleboro, Vermont, United States). When collecting FRAP data, the emission signal was filtered using a 534/30 emission filter (Chroma Technologies, Brattleboro, Vermont, United States) before being detected by a photomultiplier tube (PMT) (Hamamatsu, Bridgewater, New Jersey, United States). The PMT output was directed to a photon counter (model No. SR400; Stanford Research Systems, Sunnyvale, California, United States).

## 3 Results

### 3.1 Evaluating How the Diffusion-Convection Model Fails in the Presence of Shear

**Fig. 4:**
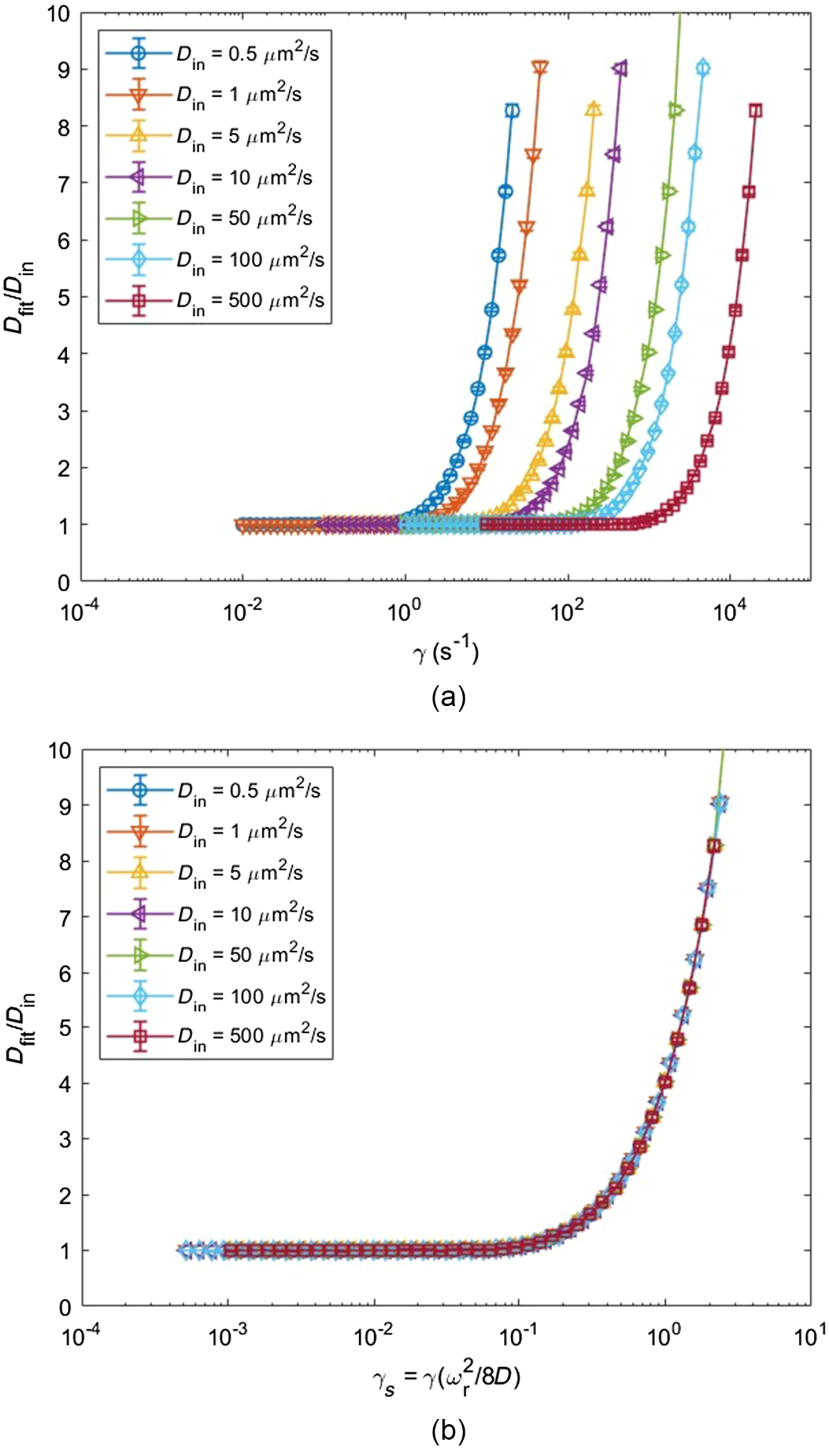
Accuracy of diffusion-convection model for various diffusion coefficients as a function of shear rate and scaled shear rate. (a) MPFRAP curves were simulated with Monte Carlo simulation for various input diffusion coefficients with a central velocity of zero ($v_{o}=0$) for various ranges of shear rate. Curves were fit to the diffusion-convection model and the fit diffusion coefficient was extracted. Data are shown as mean ± standard deviation (n=20). (b) Scaling the x-axis of (a) by the relative contribution of shear and diffusion to the fluorescence recovery, $\gamma_{s}=\gamma (\omega_{r}^{2}/8D)$, allows curves of all values of diffusion coefficient to overlay into a single curve.

To evaluate the performance of the previous diffusion-convection model in the presence of shear, we performed a series of simulations modeling fluorescence recovery with various diffusion coefficients ranging from $0.5\;\mu m^{2}/s$ to $500\;\mu m^{2}/s$, with a flow velocity in the center of the focal volume of zero ($v_{o}=0$) and with a range of shear rates from 0.01 to $10000\;s^{-1}$. These curves were fit to the diffusion-convection model and their diffusion coefficients were extracted. To determine accuracy of the fit, the fitted diffusion coefficient was divided by the input diffusion coefficient, thus an accurate fit will yield a ratio of 1. Figure 4(a) shows the accuracy of the diffusion-convection model for various diffusion coefficients as a function of shear rate. As expected, increasing shear will produce erroneously high values of measured D, to an extent that varies with the actual value of diffusion coefficient relative to shear rate. To remove the effect of diffusion coefficient, we scaled the x-axis by defining the scaled shear rate as the ratio of $\tau_{D}$ to $\tau_{\gamma }$, thus $\gamma_{s}=\gamma (\omega_{r}^{2}/8D)$. This dimensionless independent variable quantifies the relative contribution of shear and diffusion to the transport of molecules into and out of the focal volume to reveal a universal impact of shear rate on the accuracy of fit, which can be seen in Fig. 4(b). We see that the diffusion-convection model produces erroneous fits when $\gamma_{s}∼0.5$ and greater. Thus, when the recovery rate due to shear is more than half the recovery rate due to diffusion, the diffusion-convection model is no longer valid.

### 3.2 Evaluating the Impact of Scaled Shear Rate on Previous Models

Using the concept of scaled shear rate, we moved on to observing how all three models behave as scaled shear rate is varied, at three different scaled velocities. The input diffusion coefficient for these simulations was set to $60\;\mu m^{2}/s$, which is a typical diffusion coefficient for FITC-BSA. Figure 4 shows the accuracy of fit for the diffusion-only model (red), diffusion-convection model (blue), and the new shear flow model (green) over a wide range of scaled shear values, for various scaled velocities, $v_{s}=0,0.5$, and 1. Similar to the scaled shear rate, the scaled velocity is a dimensionless independent variable that quantifies the relative contribution of flow and diffusion to the transport of molecules into and out of the focal volume^10^ (i.e., $v_{s}=\tau_{D}/\tau_{v}=\omega_{r}v_{o}/8D)$.

**Fig. 5:**
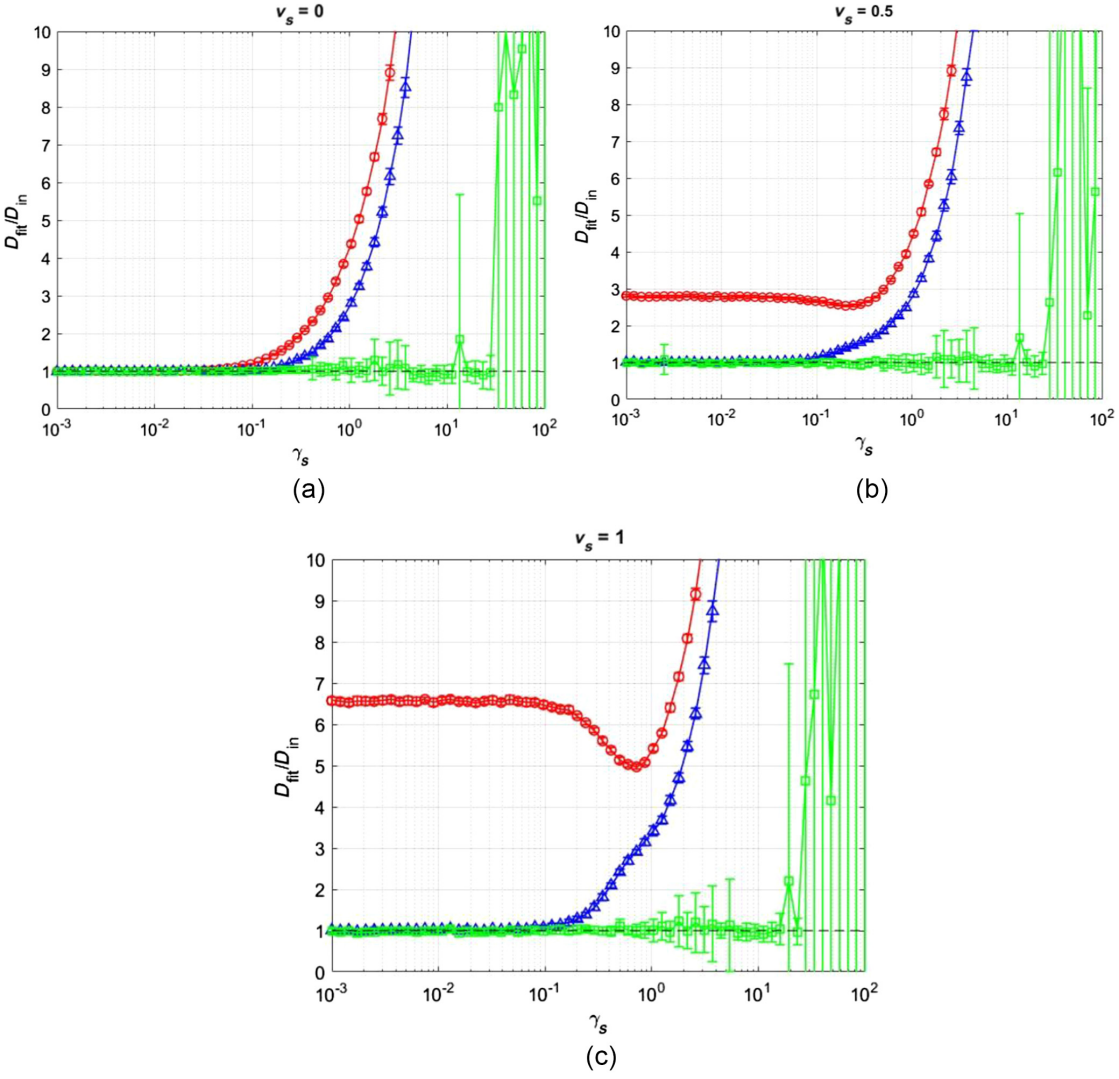
Effect of scaled shear rate on accuracy of the three fitting models. Investigating the effects of scaled shear rate on the accuracy of fitted diffusion coefficient for the diffusion-only model (red, circles), diffusion-convection model (blue, triangles), and the shear flow model (green, squares) at $v_{s}=0$ (a), 0.5 (b), and 1.0 (c). Data are shown as the mean ± standard deviation (n=20).

In Fig. 5(a), the scaled velocity is zero, representing an unphysical but mathematically attractive situation where the focal volume is placed exactly where the net transverse flow at z=0 is zero, reminiscent of a “whirlpool.” We see that all three models produce accurate diffusion coefficients up to a scaled shear rate of ∼0.3. At scaled shear rates greater than ∼0.3 and ∼0.5, respectively, we see the diffusion-only and diffusion-convection model produce erroneous values of D, while the shear flow model continues producing accurate D values up until $\gamma_{s}∼30$. In Fig. 5(b), the scaled velocity is set to 0.5 and we see the diffusion-only model produces erroneous values of D at all values of scaled shear rate, while the diffusion-convection model produces accurate diffusion coefficients up until $\gamma_{s}∼0.5$, and the shear flow model produces accurate D values up until $\gamma_{s}∼30$. In Fig. 5(c), the scaled velocity is set to 1, thus indicating an equal contribution of flow and diffusion to the fluorescence recovery in the center of the focal volume. Once again, we see the diffusion-only model producing erroneous D at all values of scaled shear rate, while the diffusion-convection model produces accurate diffusion coefficients up until $\gamma_{s}∼0.5$, and the shear flow model continues to produce accurate diffusion coefficients up until $\gamma_{s}∼30$.

### 3.3 Accuracy of Fitting Models Over Large Ranges of $v_{s}$ and $\gamma_{s}$

**Fig. 6:**
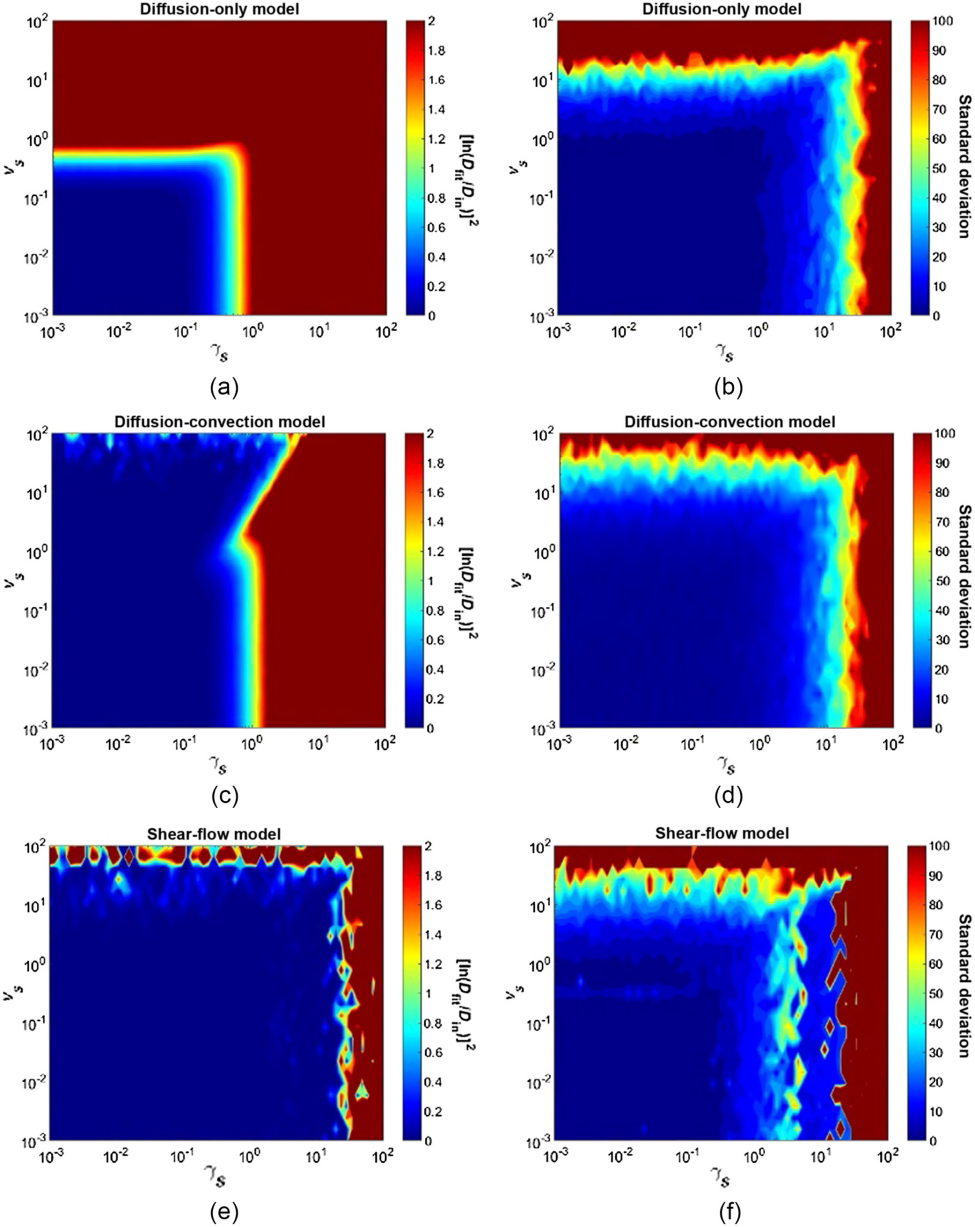
Results of three fitting models over various scaled velocities and scaled shear rates. Fluorescence recovery curves were generated in a high-throughput manner at 1728 different combinations of scaled velocities and scaled shear rates ranging from $10^{-3}$ to $10^{2}$. Curves were then fit to the diffusion-only model (a, b), diffusion-convection model (c, d), and shear flow model (e, f), and the fitted diffusion coefficient was extracted. Data are shown as the mean of (n=20). Accuracy of the resultant diffusion coefficients is shown in panels (a, c, e) by the color bar, which represents ${\left[\ln(\frac{D_{\text{fit}}}{D_{\text{in}}})\right]}^{2}$, where an accurate fit diffusion coefficient will yield a value of 0, shown as dark blue. The average standard deviation is shown in panels (b, d, f) (n=20), where smaller values are shown as dark blue.

Next, we performed Monte-Carlo simulations for a wide range of scaled velocities and scaled shear rates to visualize the area of the $v_{s}\text{-}\gamma_{s}$ parameter space over which the three models produced accurate diffusion coefficients. We preformed simulations at 27×64=1728 different combinations of scaled velocity and scaled shear rate, respectively. We then fit the resultant fluorescence recovery curves to the three models and plotted the accuracy of the fitted diffusion coefficient as contour plots in Fig. 6. In these plots, using the $D_{\text{fit}}/D_{\text{in}}$ ratio as a measure of accuracy does not visually highlight erroneous values of $D_{\text{fit}}$ that are smaller than $D_{\text{in}}$ as well as it highlights $D_{\text{fit}}$ values that are too large. To facilitate visualization of the resultant accuracy of fit, here our measure of accuracy is presented as the square of the natural log of the fit diffusion coefficient divided by the input diffusion coefficient, $[\ln(D_{\text{fit}}/D_{\text{in}}){]}^{2}$. Thus, an accurate fit diffusion coefficient ($D_{\text{fit}}=D_{\text{in}}$) will yield a value of 0, if the fit diffusion coefficient is too small and the resulting $D_{\text{fit}}/D_{\text{in}}$ is 0.1, our metric will produce a value of 5.3, and if the fit diffusion coefficient is too large and the resulting $D_{\text{fit}}/D_{\text{in}}$ is 10, our metric will produce a value of 5.3. Thus, our metric displays erroneously small values of $D_{\text{fit}}$ in a similar way as erroneously large values of $D_{\text{fit}}$.

Figure 6(a) shows the accuracy of $D_{\text{fit}}$ over the $v_{s}\text{-}\gamma_{s}$ parameter space when fit to the diffusion-only model. We can see that the diffusion-only model produces accurate diffusion coefficients for values of $v_{s}$ and $\gamma_{s}$ less than ∼0.3 (dark blue region). Figure 6(c) show the accuracy of $D_{\text{fit}}$ over the $v_{s}\text{-}\gamma_{s}$ parameter space when fit to the diffusion-convection model. As expected, we see the range of scaled velocities that produce an accurate diffusion coefficient significantly increases over that of the diffusion-only model. However, for any value of $v_{s}$, the model produces erroneous diffusion coefficients when $\gamma_{s}$ exceeds ∼0.5. Figure 6(e) shows the accuracy of $D_{\text{fit}}$ over the $v_{s}$-$\gamma_{s}$ parameter space when fit to the shear flow model. We see that the shear flow model produces accurate $D_{\text{fit}}$ values over an area much larger than the diffusion-only and diffusion-convection models and begins to produce erroneous values when $\gamma_{s}$ exceeds ∼30.

### 3.4 Improving $D_{\text{fit}}$ with *a priori* Knowledge

As mentioned previously, no bounds were imposed on any fitting parameters in the above figures to remain unbiased and test the true authenticity and rigor of the shear flow model. Thus, Fig. 6(c) shows the accuracy of the shear flow model to determine the diffusion coefficient of a system in the worst case scenario where no *a priori* knowledge is provided. However, there is usually some level of *a priori* knowledge about one’s system that can be used to improve the fitting process. Table 1 shows how the fitted diffusion coefficient changes in the absence and presence of *a priori* knowledge about one’s system for two points taken from the red region of Fig. 6(e), where all three models failed to produce an accurate diffusion coefficient. First, we consider the case where we know within a factor of ten what the possible values are for all four fitting parameters. In this case, we impose a lower bound that is one tenth of the true values of each parameter and upper bound that is ten times the true values of each parameter. This results in a fitted diffusion coefficient that is much closer to the true diffusion coefficient ($D=60\;\mu m^{2}/s$). In the best case scenario, we suppose that the velocity and shear rate of a system is known or measured independently by the user (using fluorescent beads with line scans, etc.). In this case, $\tau_{v}$ and $\tau_{\gamma }$ can be fixed and $\tau_{D}$ and β can be free fitting parameters with no bounds. We see that in this case we get a diffusion coefficient that is much more accurate than the diffusion coefficient obtained with no bounds on the fitting parameters.

**Table 1:** Fitted diffusion coefficient in the presence of different *a priori* knowledge about the system. Two regions of poor $D_{\text{fit}}$ values were chosen from Fig. 6(c): one in with high $v_{s}$ and one with high $\gamma_{s}$. The first column shows $D_{\text{fit}}$ when all four parameters are fit with no bounds [as in Fig. 5(c)], the second column shows $D_{\text{fit}}$ when a lower bound (lb) and upper bound (ub) are imposed on all four parameters, and the third column shows $D_{\text{fit}}$ when $\tau_{v}$ and $\tau_{\gamma }$ are known and $\tau_{D}$ and β are free fitting parameters with no bounds. The true diffusion coefficient is $60\;\mu m^{2}/s$. Data are shown as mean ± SEM (n=20).

	No bounds	l.b. = 0.1 × truth	Known $\tau_{v}$ and $\tau_{\gamma }$ with no bounds on $\tau_{D}$ and β
		u.b. = 10 × truth	
$v_{s}=0.2031$	$139.35\pm 428.57\;\mu m^{2}/s$	$59.72\pm 32.08\;\mu m^{2}/s$	$57.99\pm 22.46\;\mu m^{2}/s$
$\gamma_{s}=40.1028$			
$v_{s}=0.0016$	$133.09\pm 330.81\;\mu m^{2}/s$	$63.10\pm 12.42\;\mu m^{2}/s$	$63.46\pm 11.43\;\mu m^{2}/s$
$\gamma_{s}=19.31$			

### 3.5 *In vitro* MPFRAP with Shear Flow

Next, we performed MPFRAP in an *in vitro* system which allowed for various combinations of $v_{s}$ and $\gamma_{s}$ with a solution of 2000 kDa FITC-dextran with a known diffusion coefficient. The velocity of the solution through the channel was manipulated by adjusting the height of the inlet reservoir. To determine the shear rate at various locations within the channel, the velocity measured with line scan images was plotted as a function of height within the channel and fit to a second-order polynomial (Fig. 3); the shear rate as a function of height within the channel was computed as the derivative of the velocity profile, as shown in Fig. 3.

**Fig. 7:**
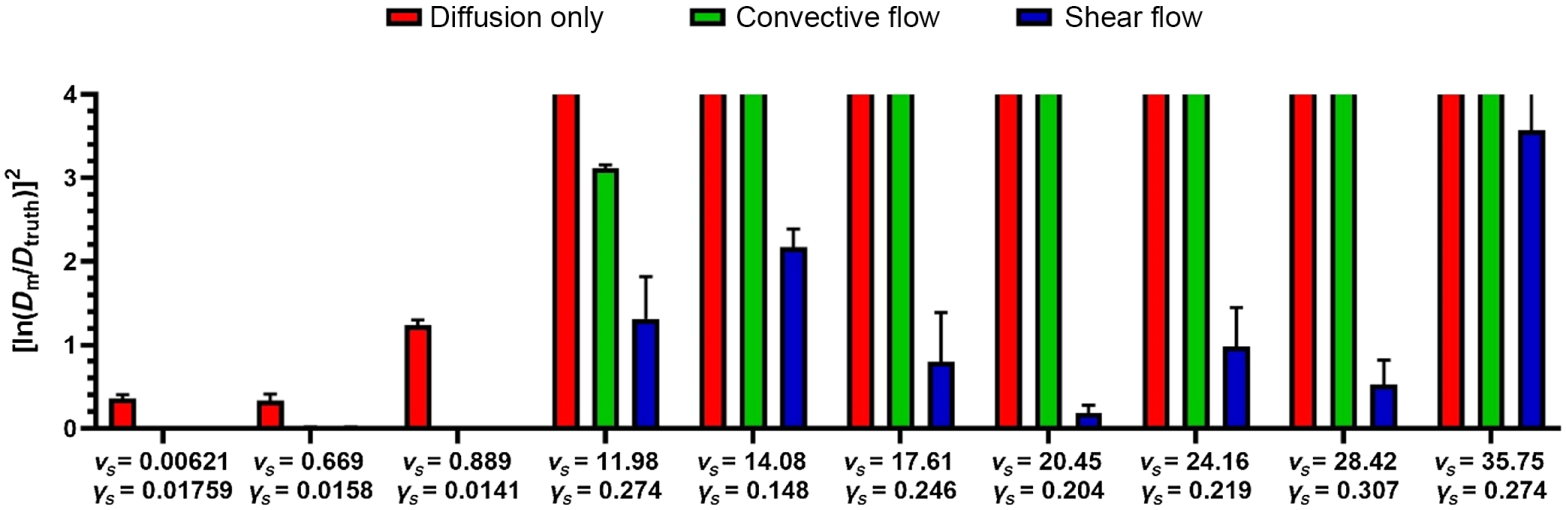
Accuracy of the three MPFRAP models in determining D *in vitro* in the presence of various combinations of flow and shear rate. Scaled velocity and shear rates were measured as described in the methods section. Accuracy of $D_{m}$ is determined by calculating $[\ln(\frac{D_{m}}{D_{\text{truth}}}){]}^{2}$, which will yield values closer to zero when $D_{m}$ is closer to $D_{\text{truth}}$. Data are shown as mean (n=3) ± standard deviation. Note that bars for convective flow and shear flow in the first three columns are not visible because their mean values and error bars are so close to zero, while the bars for diffusion only and convective flow for the later columns are cut off, to keep the detailed behavior of the shear flow model visible.

Multiple MPFRAP measurements were taken immediately after line scan acquisition at a specific height within the channel. The measured diffusion coefficient ($D_{m}$) was then compared to the known diffusion coefficient to determine the accuracy of the three MPFRAP models *in vitro*. First, we performed MPFRAP in a droplet of FITC-dextran to check that our system could accurately determine the diffusion coefficient in a diffusion-only system, this yielded a diffusion coefficient of $9.8\;\mu m^{2}/s$ which is comparable to values reported in the literature;^20^ this measured diffusion coefficient ($D_{\text{truth}}$) was used as our ground truth value that all other measurements were compared to. All MPFRAP curves were fit to the three MPFRAP models with the same criteria as the simulated MPFRAP curves with no *a priori* bounds imposed, (representing the “worst case” scenario), as shown in Fig. 7.

## 4 Discussion

Here we derived an improved model of MPFRAP that can more accurately measure diffusion coefficients in systems where shear flow is present. Previous MPFRAP models were designed to only calculate diffusion coefficients in systems with no flow (diffusion-only), and flow with no shear stress (diffusion-convection). We began by observing how the previous diffusion-convection model begins to fail, which is shown in Fig. 4: simulations were performed for multiple input diffusion coefficients in the absence of central flow ($v_{o}=0$) over a large range of shear rates γ. For each simulated diffusion coefficient $D_{\text{in}}$, we see erroneous $D_{\mathrm{fit}}$ are exponentially produced at some shear rate which is different for different values of $D_{\text{in}}$. To observe a universal relationship between erroneous $D_{\text{fit}}$ and shear rate, we scaled the x-axis of Fig. 4(a) by the relative contribution of shear and diffusion to the overall fluorescence recovery, thus the scaled shear rate in Fig. 4(b) is $\gamma_{s}=\gamma (\omega_{r}^{2}/8D)$. This dimensionless independent variable allowed all the curves in Fig. 4(a) to overlap into a single curve, where we can now see that the diffusion-convection model begins to produce erroneous fits at $\gamma_{s}∼0.5$. A scaled shear rate of 0.5 indicates that the recovery rate due to shear is about half the recovery rate due to diffusion. Thus, at low values of scaled shear, we expect accurate $D_{\text{fit}}$ values because the recovery of the curve is dominated by diffusion. At high values of scaled shear, we expect erroneous values of $D_{\text{fit}}$ because the recovery of the curve is dominated by shear and the diffusion-convection model has no mechanism to account for shear.

With the concept of scaled shear rate now defined, we moved on to observing how all three models behave in the presence of scaled shear rate at three different values of scaled velocities, shown in Fig. 5. The scaled velocity is the relative contribution of flow and diffusion to the overall fluorescence recovery within the focal volume, i.e., $v_{s}=v_{o}(\omega_{r}/8D)$, and has been described previously.^10^ In Fig. 5(a), we observe the accuracy and precision of the three models in the case $v_{s}=0$ over a range of scaled shear rates. We see that the diffusion-only model begins to produce erroneous $D_{\text{fit}}$ values at $\gamma_{s}∼0.3$. Interestingly, we see increasingly erroneous $D_{\text{fit}}$ values that have consistently small error bars, which illustrates the difference between precision and accuracy. Since the diffusion-only model has no mechanism to account for recovery due to shear, it assigns all recovery to diffusion. This effect produces very inaccurate fits but will produce consistently similar (and poor) fits. We also observe the same phenomenon with the diffusion-convection model in Fig. 5(a), with some improvement in the range of accuracy of $D_{\text{fit}}$ compared to the diffusion-only model. The new shear stress model significantly improves the range of accuracy of $D_{\text{fit}}$ and starts to produce erroneous fits at $\gamma_{s}∼30$. Since this new model explicitly considers shear flow, its mode of failure is different from the previous two models. The model can correctly assign shear-based recovery to the shear parameter γ, maintaining accuracy in the fitted D at higher shear rates, up to $\gamma_{s}∼30$. However, it loses accuracy and precision in the fitted diffusion coefficient as $\gamma_{s}$ exceeds ∼30. The recovery becomes dominated by high shear and the actual physical contribution of diffusion to the recovery process becomes insignificant, which explains the loss of accuracy. Additionally, there are three variables that recovery can be assigned to and this can be accomplished with several combinations of these variables, which explains the loss in precision. Nonetheless, the shear flow model significantly improves the range of accuracy of $D_{\text{fit}}$ over various shear rates compared to the diffusion-only and diffusion-convection models.

The limits of accuracy of $D_{\text{fit}}$ for all three models can be seen in Fig. 6, where the accuracy of $D_{\text{fit}}$ is plotted as a function of 1728 different combinations of scaled velocity and scaled shear rate. Figure 6(a) shows the accuracy of $D_{\text{fit}}$ produced by the diffusion-only model over this $v_{s}\text{-}\gamma_{s}$ parameter space, and we can see an accurate diffusion coefficient (dark blue) is produced for a relatively small area of this parameter space, bounded by $v_{s}∼0.3$ and $\gamma_{s}∼0.3$. Thus, when the recovery rate due to flow and shear is <0.3 times the recovery time due to diffusion, the diffusion-only model is accurate. Figure 6(b) shows the standard deviation of $D_{\text{fit}}$, which remains low even in much of the regions of poor $D_{\text{fit}}$, suggesting that the diffusion-only model is still precise in regions where it is no longer accurate, as discussed above.

The diffusion-convection model expands the area of parameter space over which accurate diffusion coefficients are produced in the direction of increasing $v_{s}$, as seen in Fig. 6(c). The limits of accurate diffusion coefficients for the diffusion-convection model are $v_{s}∼100$ and $\gamma_{s}∼0.5$, which is a considerable improvement in one direction of parameter space ($v_{s}$) compared to the diffusion-only model. Fig. 6(d) shows the standard deviation of $D_{\text{fit}}$, which reveals two features of the diffusion-convection model; it remains accurate but less precise at high values of $v_{s}$, and becomes inaccurate and remains precise as $\gamma_{s}$ increases.

Finally, the shear flow model shown in Fig. 6(e) significantly expands the area over which accurate diffusion coefficients are produced in the direction of increasing $\gamma_{s}$, with accurate values of D possible up to scaled shear rates of $\gamma_{s}∼30$. Figure 6(f) shows the standard deviation of $D_{\text{fit}}$ produced by the shear-flow model. Unlike the previous two models, the bounds of precision for the shear-flow model are almost the same as the bounds of accuracy. Additionally, we see the model remains accurate at higher values of $v_{s}$, but is slightly less precise than the diffusion-convection model. This is most likely due to the fitting algorithm having the flexibility of assigning the fluorescence recovery to either $\tau_{v}$ or $\tau_{\gamma }$ in the shear model. Of course, these exact errors in $D_{\text{fit}}$ are a function of the particular choices of β (0.6), relative noise (3%), and focal volume dimensions (NA = 0.8) chosen for our simulations and while we expect the same qualitative relationships between $v_{s}$, $\gamma_{s}$, and fitting model accuracies for other choices, the quantitative relationships may change.

In the absence of any *a priori* knowledge, the shear flow model covers a much larger area in $v_{s}\text{-}\gamma_{s}$ parameter space than previous models and begins to produce erroneous diffusion coefficients on the outer border of this parameter space. However, in the presence of *a priori* knowledge, we see a noticeable improvement in the fitted diffusion coefficient in Table 1. Imposing even weak bounds on all four fitting parameters increases the efficacy and accuracy of the fitting algorithm, thus improving the fitted diffusion coefficient. In the best case scenario, independently knowing the velocity and shear rate of one’s system will improve the fitted diffusion coefficient even more since the number of fitting parameters decreases from four to two.

Finally, we explored the accuracy of all three fitting models on *in vitro* FRAP data collected in a microfluidic channel with various combinations of velocity and shear rate. The velocities and shear rates at which we collected our measurements are within the bounds of the axes in Fig. 6. As seen in Fig. 7, the trends are the same as in the simulations: the diffusion only model produces relatively accurate diffusion coefficients at very low values of velocity and shear rate and produces increasingly erroneous diffusion coefficients as velocity and shear rate increase. We also see the diffusion-convection model retains accuracy at higher shear values than the diffusion only model, but also begins to fail in the presence of increasing shear rate. Finally we see the same for the shear-flow model at the first three combinations, and we see the metric of error remain much smaller than the other models as the velocity and shear rate increase. Again, the exact values of $v_{s}$ and $\gamma_{s}$ at which each model begins to lose accuracy will vary with several experimental parameters including β, relative noise, etc. It is important to note that no bounds were imposed on the fitting of these curves. According to our discussion accompanying Table 1, we expect the accuracy of the shear flow model to improve even more when the appropriate bounds are imposed.

There are numerous biological applications where the new shear flow fitting model can provide useful information on diffusion coefficients by expanding the region of $v_{s}\text{-}\gamma_{s}$ parameter space that is accessible for accurate diffusion coefficients, relative to previous fitting models. In the past, our previous diffusion-convection model has been used to reevaluate the hypothesis that aquaporin-4 water channels facilitate convective transport of solutes within brain parenchyma by performing MPFRAP on various solutes moving through mouse brain.^21^ This model has also been used to measure lymph viscosity (via measurement of D) in lymphatic vessels afferent to popliteal lymph nodes in the hind footpad of mice.^6^ Looking forward, there are many combinations of flow speed and shear rate values that occur in biological systems, such as microvessels and artificial organ constructs, which occur within the region of $v_{s}\text{-}\gamma_{s}$ parameter space now accessible with the new model.^13^^–^^16^

This improved model of MPFRAP can also be helpful in the field of microfluidics, which includes devices as diverse as organ-on-a-chip tumor models and point-of-care analytic devices in low resource settings.^22^^,^^23^ A variant of MPFRAP has been used to validate the simulated interstitial flow and diffusion within a microfluidic device that controls cellular communication mediated by interstitial flow.^24^ Shear is a salient feature of the flow profiles in narrow microfluidic channels and has been exploited in numerous such devices to stimulate biological responses in cells.^25^^,^^26^ Due to their small size, the aqueous flow in microfluidic devices is typically laminar, and diffusion is a prominent process by which solutes can be separated.^27^^,^^28^ For example, a T-sensor is a diagnostic microfluidic device for chemical assays which utilizes the diffusion of solutes to extract information about the solute molecular weight and concentration.^27^^–^^30^ Hence, we see that a fitting model for MPFRAP that produces accurate diffusion coefficients in the presence of shear flow may contribute in the area of microfluidics as well as in flow in biological systems.

## Code, Data, and Material Availability

The code used to generate and fit simulated data can be found on Code Ocean: https://doi.org/10.24433/CO.4821156.v1. Data generated in this study are available from the authors upon request.
